# Estrogen and estrogen receptor alpha promotes malignancy and osteoblastic tumorigenesis in prostate cancer

**DOI:** 10.18632/oncotarget.6317

**Published:** 2015-10-31

**Authors:** Sweta Mishra, Qin Tai, Xiang Gu, James Schmitz, Ashley Poullard, Roberto J. Fajardo, Devalingam Mahalingam, Xiaodong Chen, Xueqiong Zhu, Lu-Zhe Sun

**Affiliations:** ^1^ Department of Cellular and Structural Biology, University of Texas Health Science Center, San Antonio, Texas, USA; ^2^ Department of Orthopedic Surgery, University of Texas Health Science Center, San Antonio, Texas, USA; ^3^ Cancer Therapy and Research Center, University of Texas Health Science Center, San Antonio, Texas, USA; ^4^ Dental School, University of Texas Health Science Center, San Antonio, Texas, USA; ^5^ Department of Obstetrics and Gynecology, the Second Affiliated Hospital of Wenzhou Medical University, Wenzhou, China; ^6^ Department of Medicine, Brown University, Providence, Rhode Island, USA; ^7^ Department of Vascular Surgery, The Second Xiangya Hospital and Xiangya School of Medicine, Central South University, Changsha, Hunan, China

**Keywords:** prostate cancer, estrogen, estrogen receptor, bone, osteoblastic, metastasis

## Abstract

The role of estrogen signaling in regulating prostate tumorigenesis is relatively underexplored. Although, an increasing body of evidence has linked estrogen receptor beta (ERβ) to prostate cancer, the function of estrogen receptor alpha (ERα) in prostate cancer is not very well studied. We have discovered a novel role of ERα in the pathogenesis of prostate tumors. Here, we show that prostate cancer cells express ERα and estrogen induces oncogenic properties in prostate cancer cells through ERα. Importantly, ERα knockdown in the human prostate cancer PacMetUT1 cells as well as pharmacological inhibition of ERα with ICI 182,780 inhibited osteoblastic lesion formation and lung metastasis *in vivo*. Co-culture of pre-osteoblasts with cancer cells showed a significant induction of osteogenic markers in the pre-osteoblasts, which was attenuated by knockdown of ERα in cancer cells suggesting that estrogen/ERα signaling promotes crosstalk between cancer and osteoblastic progenitors to stimulate osteoblastic tumorigenesis. These results suggest that ERα expression in prostate cancer cells is essential for osteoblastic lesion formation and lung metastasis. Thus, inhibition of ERα signaling in prostate cancer cells may be a novel therapeutic strategy to inhibit the osteoblastic lesion development as well as lung metastasis in patients with advanced prostate cancer.

## INTRODUCTION

Prostate cancer is the second leading cause of cancer-related death in the United States with an estimated 233,000 new cancer cases and 29,480 deaths reported in 2014 [1]. Given the widely recognized role of androgens in the development and progression of prostate cancer, androgen ablation therapy is the mainstay for the treatment of metastatic prostate cancer. However, most patients will eventually develop androgen-independent prostate cancer, highlighting an urgent need for the alternative treatment strategies. Although the growth and differentiation of prostate gland is primarily controlled by androgens, estrogens are also hormonal risk factors in the development of benign prostatic hyperplasia and prostate cancer [2, 3]. Although estrogen therapy was historically used to reduce the androgen levels in men with advanced prostate cancer [4], more recent studies have also shown its involvement in prostate carcinogenesis. For example, estrogen can induce neoplastic epithelial morphology in both human and rat prostates and regulated prostate specific gene expression [5, 6]. Prolonged treatment of rodents with a high combined dose of androgen and estrogen induces stromal hypertrophy, epithelial dysplasia and enlargement of the prostate gland [7, 8]. Studies have suggested that anti-estrogens inhibit the development and progression of prostate cancer under experimental and clinical conditions [9, 10]. Some of these anti-estrogen compounds are in clinical trials to study their efficacy in prostate cancer prevention [11, 12]. The combination therapy of an estrogen receptor antagonist, tamoxifen with an anti-androgen bicalutamide, reduced gynecomastia and breast pain in prostate cancer patients receiving anti-androgen therapy in a multicenter trial [13]. Recently, a phase III clinical trial with a selective estrogen receptor modulator Toremifene, showed a significant reduction in new vertebral fractures in men with prostate cancer receiving androgen ablation therapy [11, 14]. Thus, understanding the oncogenic role of estrogen signaling in prostate carcinogenesis might provide a new therapeutic avenue for treating patients with advanced prostate cancer.

The presence of estrogen receptors (ER) in prostate suggests that estrogens may act directly in prostate epithelial cells [15]. The two ER subtypes, ERα and ERβ, have different expression patterns with several studies presenting conflicting results in their expression as well as function during prostate carcinogenesis. ERβ is regarded as the predominant subtype in majority of the epithelial cells, as well as in some stromal cells of the prostate. ERβ is considered to be tumor suppressive in many cancers including the prostate [16–19]. ERβ can cause anti-proliferative effects as well as apoptosis in the castration-resistant basal epithelial cells in the prostates of aromatase knockout mice [20, 21]. Anti-estrogens can inhibit tumor growth through ERβ-mediated regulation of the tumor-suppressing transcription factor KLF5 in prostate cancer cells [22]. However, a study by Zellweger et al. showed that ERβ expression and AR phosphorylation correlated with poor clinical outcome in hormone-naïve prostate cancer and found increased ERβ in castration-resistant phase of the disease [23]. The study by Leav et al. showed a reappearance of ERβ expression in bone and lymph node metastasis, suggesting a causal link to the development of late stage disease [24]. A recent study showed that the ERβ splice variant 2 (ERβ2) had oncogenic properties and was involved in osteolytic bone metastasis in strong contrast to the tumor suppressing effects of the other isoform ERβ1 [25]. Thus, differentiating the functions of splice variants for estrogen receptors will further elucidate our understanding of their roles in prostate cancer progression. While the role of ERβ in prostate cancer is well studied, there are relatively few studies on the functional role of ERα in prostate tumorigenesis. Estrogen was shown to mediate prostate cancer progression through ERα in a genetic knockout mouse model for both ERα and ERβ receptors [26]. While the prostates of wild type and ERβ knockout mouse showed hyperplasia and PIN (prostatic intra-epithelial neoplasia) lesions when treated with testosterone and estrogen for four months, the prostates of ERα knockout animal did not develop these pathologies [26]. Furthermore, high ERα mRNA and protein levels were detected in hormone refractory and metastatic lesions with lymph node and bone metastatic samples [27]. However, the exact role of ERα in prostate tumorigenesis and formation of metastatic lesions has not been extensively investigated.

More than 80% of prostate cancer patients develop bone metastasis in the advanced stages of the disease [28]. The types of lesions that patients develop are predominantly osteoblastic or bone forming. The mechanism for the osteoblastic lesion development is poorly understood partly due to a lack of suitable model systems that recapitulates bone formation in the prostate cancer patients. The transgenic PTEN knockout mouse models have only a 27% bone metastasis incidence rate [29]. Prostate cancer cell lines such as PC-3, LNCaP, LuCaP, and LAPC-9 develop osteolytic or mixed lesions in immunocompromised mouse xenograft models [30]. We have shown previously that the human prostate cancer PacMetUT1 cell line induced extensive bone formation *in vivo* and could serve as a useful model for investigating the mechanism of osteoblastic lesion formation [31]. In this study, we have investigated the oncogenic roles of estrogen and ERα in various prostate cancer cell lines including PacMetUT1. Our results suggest that estrogen-induced osteoblastic bone formation and lung metastasis is mediated through ERα. Thus, targeting ERα in prostate cancer patients with advanced metastatic disease might be a novel and efficient therapeutic strategy to reduce bone lesions and lung metastasis.

## RESULTS

### Prostate cancer cells express ERα and are estrogen responsive

**Figure 1:**
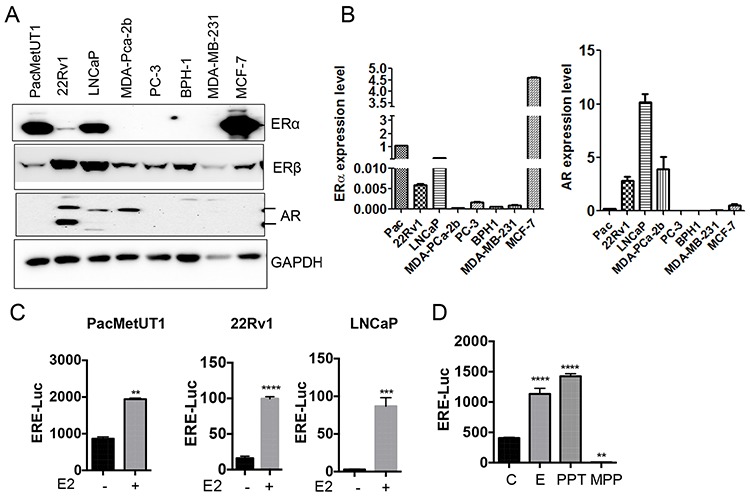
Prostate cancer cells are estrogen responsive **A** and **B.** Cell lysates and cDNAs were used for ERα, ERβ and AR expression analysis in different prostate cancer cell lines and the benign prostatic hyperplasia (BPH-1) cell line by Western blot and real-time RT-PCR respectively. The mRNA level of ERα and AR from real-time RT-PCR assays were normalized with the mRNA level of actin in each sample and presented as mean ± sem from triplicate measurements. **C.** Estrogen responsiveness of prostate cancer cells was assessed using ERE-Luciferase reporter assay. **P < 0.01, ***P < 0.001 and ****P < 0.0001 with two-tailed student's *t*-test. **D.** ERE-luciferase assay in PacMetUT1 cells with estrogen and ERα agonist PPT and antagonist MPP. β-galactosidase normalized luciferase activity in both Panels C and D is presented as mean ± sem from triplicate measurements. E2 and E have been used interchangeably. **P < 0.01 and ****P < 0.0001 with one-way ANOVA analysis.

The effects of estrogen are mediated by the intracellular estrogen receptors (ERs), which regulate transcription through binding to specific DNA sequences called EREs (estrogen response elements) in the promoter regions of their target genes. When we checked the ERα, ERβ and AR status in different prostate cancer cells as well as in a benign prostatic hyperplasia cell line (BPH-1), we detected ERα protein in some prostate cancer cells and ERβ protein in all cell lines (Figure 1A and supplementary Figure 1A) even though ERα mRNA was detected in all the cells tested (Figure 1B). AR expression showed expected results with 22Rv1, LNCaP, and MDA-PCa-2b known to be AR positive, a moderate expression in PacMetUT1, and no expression in PC-3 and BPH-1 (Figure 1A and 1B). These cells were also responsive to estrogen as measured with the estrogen-responsive ERE-luciferase assay (Figure 1C). The responsiveness to estrogen was in part mediated by ERα as ERα agonist PPT also stimulated luciferase activity whereas ERα antagonist MPP reduced the basal luciferase activity driven by the ERE promoter (Figure 1D).

### Estrogen increases prostate cancer cell growth

**Figure 2:**
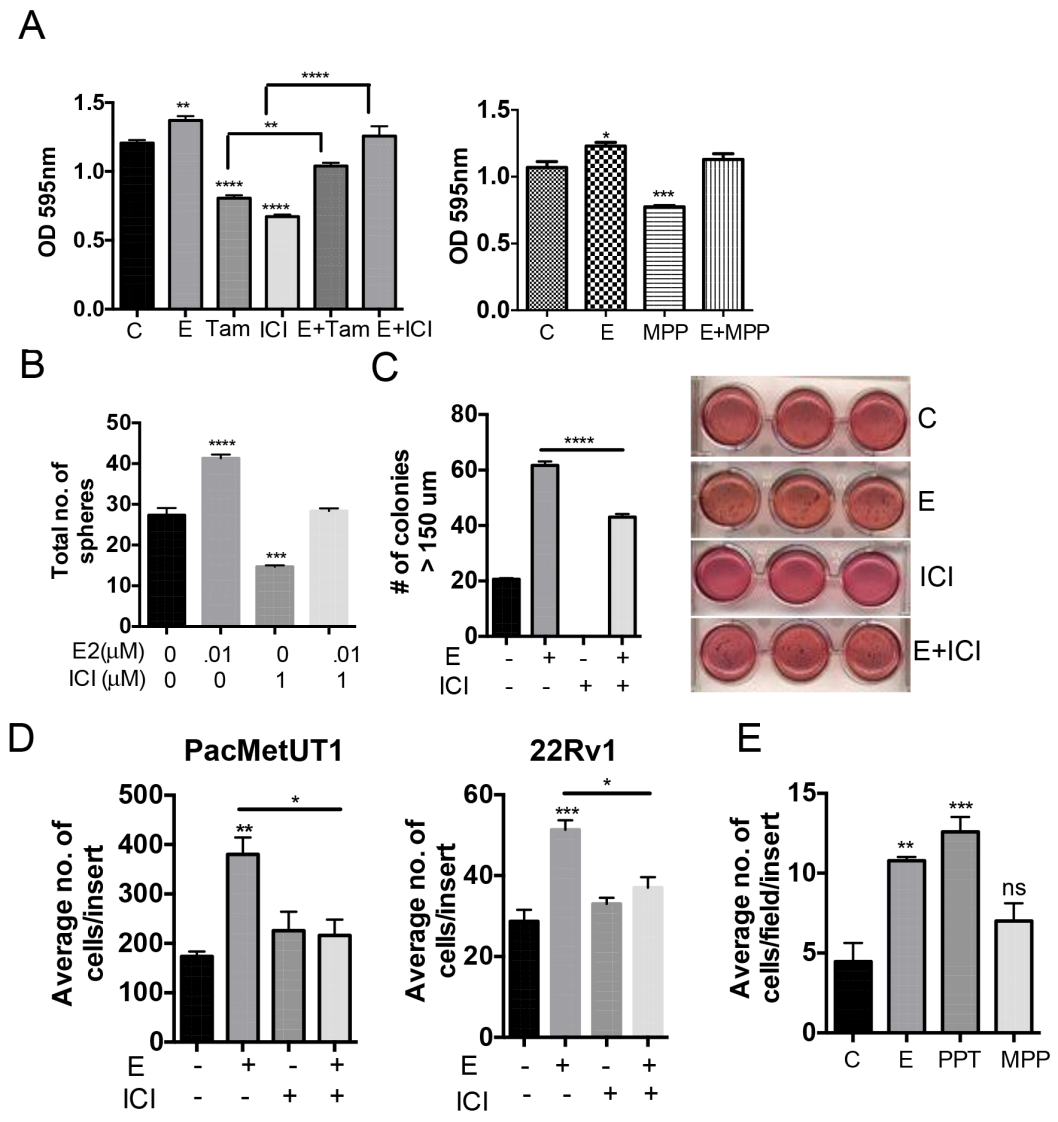
Estrogen increases anchorage-dependent and independent cell growth, migration and growth in suspension culture of prostate cancer cells **A.** PacMetUT1 cells (1,000 cells/well) were treated with estrogen (10 nM), ICI (100 nM), Tamoxifen (100 nM), or in combination for 5 days. Cell growth was analyzed by MTT assay. Data presented are mean ± sem from five measurements. PacMetUT1 cells (1,000 cells/well) were treated with estrogen (10nM) and MPP (100nM), or in combination for 5 days. Cell proliferation was checked with MTT assay from six measurements. *P < 0.05, **P < 0.01, ***P < 0.001 and ****P < 0.0001 with one-way ANOVA analysis. **B.** PacMetUT1 cells (1,000 cells/100 μL) were plated in Epicult basal medium in a 96-well low attachment plate. Cells were treated at the time of plating with estrogen (0.01 μM), ICI (1 μM), or in combination. After 48 h, colonies were quantified and presented as mean ± sem from triplicate wells. ***P < 0.001 and ****P < 0.0001 with one-way ANOVA analysis. **C.** Anchorage-independent growth of PacMetUT1 treated with estrogen (10 nM), ICI (1 μM), or in combination was measured after 7 days of growth in soft agar. Figures presented are from triplicate measurements with mean ± sem. ****P < 0.0001 with one-way ANOVA analysis. **D.** Migration of PacMetUT1 and 22Rv1 cells after treatment with estrogen (10nM), ICI (100nM) and in combination were counted in whole inserts after 18 h. Data presented are mean ± sem from triplicate wells. *P < 0.05, **P < 0.01 and ***P < 0.001 with one-way ANOVA analysis. **E.** Migration of PacMetUT1 cells after treatment with estrogen (10nM), PPT and MPP (100nM) after 18 h were counted in five high power fields (HPF) per well. Data presented are mean ± sem from triplicate wells. *P < 0.05, **P < 0.01 and ***P < 0.001 with one-way ANOVA analysis. ns denotes no statistical difference.

To check the effect of estrogen on cell growth, we treated PacMetUT1 with estrogen and two different ER antagonists, tamoxifen and ICI 182,780 (ICI) respectively. Estrogen significantly increased the cell growth after 5 days of treatment (Figure 2A and supplementary Figure 1B). Both tamoxifen and ICI decreased the cell growth, which was reversed by the addition of estrogen (Figure 2A) suggesting the functional involvement of ERs in the regulation of PacMetUT1 cell growth. Furthermore, using an ERα specific antagonist, MPP also reduced the cell growth that was regained by the addition of estrogen (Figure 2A). To determine the role of estrogen signaling in regulating the malignant properties of prostate cancer cells, we also examined its effect on anchorage-independent growth and cell migration. Estrogen significantly increased the number of spheres formed by PacMetUT1 cells in suspension culture (Figure 2B) and soft agar colonies (Figure 2C), which were antagonized by ICI. Estrogen also significantly increased the cell migration of both PacMetUT1 and 22Rv1 cells (Figure 2D) and in C4–2 cells (another bone metastatic cell line; Supplementary Figure 1C). Interestingly, unlike its effect on cell growth, the treatment with ICI itself had no effect on the basal level of cell migration (Figure 2D). This could be due to the shorter time treatment with ICI for the migration assay (16 hr of treatment) than for the growth assays (5 days on plastic, 2 days in suspension culture, or 7 days in soft agar) and/or insufficient basal level of estrogen in the system. Nevertheless, the treatment with ICI did abrogate the effect of exogenous estrogen-induced cell migration (Figure 2D). Consistently, treatment with ERα agonist PPT significantly increased the migration of PacMetUT1 whereas ERα antagonist MPP showed no effect (Figure 2E) as was observed with ICI (Figure 2D). These results again indicate the involvement of ERα in driving the malignant properties of prostate cancer cells.

### Estrogen/ERα signaling induces epithelial-to-mesenchymal transition

**Figure 3:**
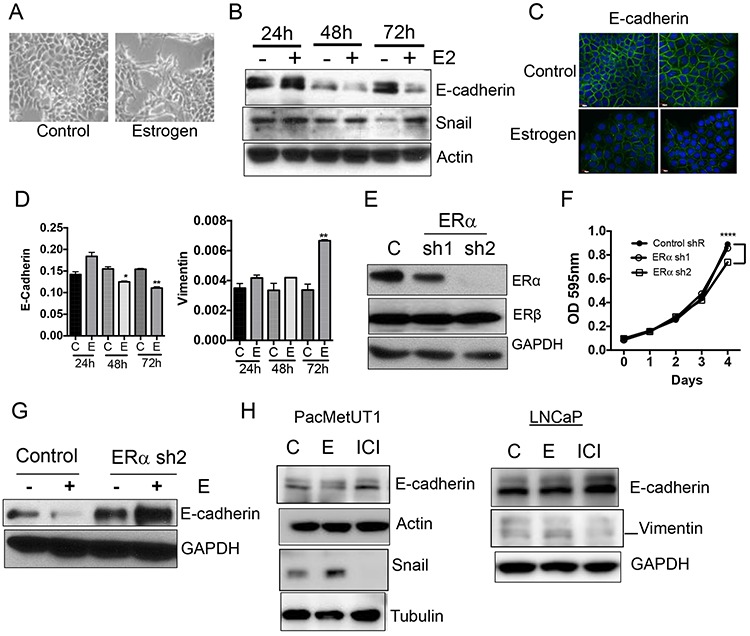
Estrogen/ERα induces epithelial-to-mesenchymal (EMT) transition **A.** PacMetUT1 cell morphology change with estrogen (100 nM) treatment for 72 hours. **B.** EMT markers, E-cadherin and Snail, were detected in PacMetUT1 cells after 24, 48 and 72 hours of treatment with estrogen (100 nM) by Western blot. **C.** Immunocytochemical staining of E-cadherin in the control and estrogen-treated (100 nM for 72 h) PacMetUT1 cells. **D.** Relative mRNA levels of E-cadherin and Vimentin in PacMetUT1 cells after 24, 48 and 72 hours of treatment with estrogen (100 nM). Data are presented as mean ± sem from three measurements with real time RT-PCR assays. * *P* < 0.05 and ** *P* < 0.01 with two-tailed student's *t*-test. **E.** ERα protein expression was stably knocked down in PacMetUT1 with two different shRNA's (denoted as sh1 and sh2 in the figure). **F.** Control and ERα knockdown PacMetUT1 cells were plated in 96-well plates at 1,000 cells/well. Their growth at the indicated time points was measured with MTT assay. Data presented are mean ± sem from quadruplicate measurements. ****P < 0.0001 with one-way ANOVA. **G.** E-cadherin protein in the control and ERα knockdown PacMetUT1 cells was measured with Western blot after 72 hours of estrogen (100 nM) treatment. **H.** E-cadherin, Snail, and Vimentin protein levels were measured in the lysates of PacMetUT1 and LNCaP cells after 72 hours of estrogen (100 nM) and ICI (1 μM) treatment. Both short and long time exposures with Snail are shown. * *P* < 0.05, **P < 0.01 and ***P < 0.001.

Epithelial-to-mesenchymal transition (EMT) is a developmental process involved in cell differentiation, migration, and morphogenesis. During tumor progression, local microenvironmental factors in a primary tumor activate the EMT program in cancer cells, which triggers tumor cell invasion [32]. We noticed a change in morphology of PacMetUT1 cells with estrogen resembling the EMT process (Figure 3A). This was confirmed by a down-regulation in E-cadherin expression and an increase in Snail upon estrogen treatment (Figure 3B–3C). There was a decrease in E-cadherin mRNA expression after both 48 and 72 hours of treatment with estrogen, and an upregulation in Vimentin mRNA level, which is a mesenchymal marker (Figure 3D). These results indicate that estrogen signaling can stimulate EMT of the prostate cancer cells, likely through ERα as ERβ was shown to inhibit EMT in earlier studies [33]. To test this hypothesis, we stably knocked down ERα in PacMetUT1 with two different shRNA sequences delivered by a lentiviral vector. As shown in Figure 3E, shRNA2 was more effective in knocking down ERα expression than shRNA1. Thus, shRNA2-transfected PacMetUT1 was used in subsequent experiments. The level of ERβ was not altered when ERα was knocked down. ERα knockdown with shRNA2 moderately reduced the growth rate of PacMetUT1 cells when compared to the control and shRNA1 cells (Figure 3F). Interestingly, estrogen mediated down-regulation in E-cadherin was completely abrogated in ERα knockdown PacMetUT1 cells (Figure 3G). Instead, we observed an induction in the basal level of E-cadherin with ERα knock down (Figure 3G). Similar effects were observed in both PacMetUT1 and LNCaP cells treated with ICI, which increased E-cadherin and decreased Snail or Vimentin (Figure 3H). All these results confirm the involvement of ERα signaling in estrogen-induced EMT.

### Estrogen/ERα signaling induces osteoblast-like features in prostate cancer cells

**Figure 4:**
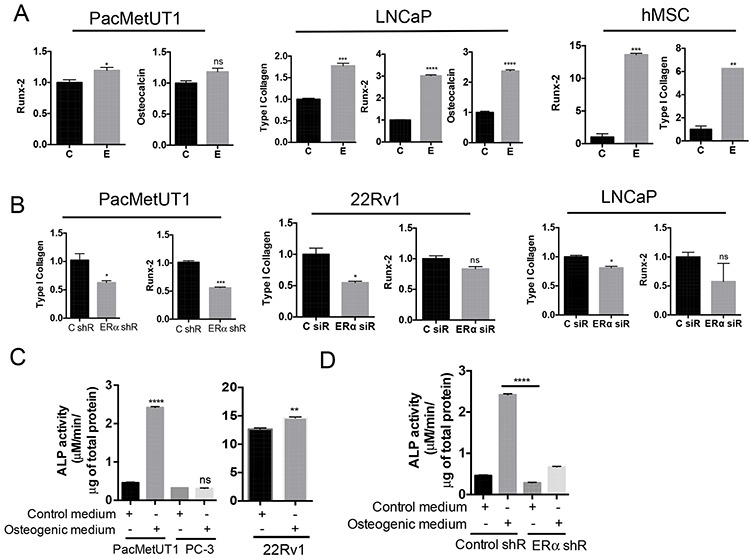
Estrogen/ERα signaling induces osteoblast-like properties in prostate cancer cells **A.** Estrogen induces osteogenic markers determined by real-time RT-PCR in PacMetUT1, LNCaP and hMSC cells when cultured in osteogenic differentiation medium for 5 days. **B.** ERα knockdown in prostate cancer cells reduces osteogenic marker expression in cells as determined by real-time RT-PCR analysis. *P < 0.05, **P < 0.01, ***P < 0.001 and ****P < 0.0001 with one-way ANOVA analysis. **C.** Alkaline phosphatase activity assay was performed in the lysate of PacMetUT1, PC-3 and 22Rv1 cells after 4 days of culture in the presence and absence of osteogenic differentiation medium. Results are normalized to total protein concentration. **D.** Alkaline phosphatase activity was measured in control and ERα knockdown PacMetUT1 cells in the presence and absence of osteogenic differentiation medium. Data are presented as mean ± sem from three measurements for the alkaline phosphatase assays. *P < 0.05, **P < 0.01, ***P < 0.001 and ****P < 0.0001.

Because estrogen/ERα signaling induced EMT and is known to promote mesenchymal stem cell differentiation to osteoblasts and bone formation (our unpublished data, [34, 35], we hypothesized that estrogen/ERα signaling may induce prostate cancer cells to become bone-forming osteoblast-like cells, a phenomenon widely reported by others as osteomimicry phenotype [36, 37]. We found that, similar to the osteoblast precursor -human mesenchymal stem cells (hMSC), estrogen upregulated osteoblastic marker expression (Runx-2, Type I Collagen, and Osteocalcin) in prostate cancer cells when cultured in osteogenic differentiation medium (Figure 4A), but not in the regular proliferation medium (Supplementary Figure 1D). Conversely, stable knockdown of ERα in PacMetUT1 cells with shRNA and a transient knockdown of ERα in 22Rv1 and LNCaP with siRNA significantly reduced the expression of osteogenic markers (Figure 4B). Culture of PacMetUT1 and 22Rv1 cells in osteoblastic differentiation medium increased the activity of alkaline phosphatase, another marker of osteoblast differentiation (Figure 4C). In contrast, the alkaline phosphatase activity did not change in PC-3 cells (Figure 4C), which does not express ERα and induces osteolytic lesions [25]. Knockdown of ERα in PacMetUT1 attenuated the induction of the alkaline phosphatase activity in osteogenic medium (Figure 4D). These results suggest that prostate cancer cells have osteoblast-like properties as they express osteogenic markers that are further elevated when cultured in osteogenic medium and stimulated by estrogen signaling through ERα. They are consistent with the published reports describing osteomimicry in prostate cancer cells [36, 37].

### ERα knockdown inhibits osteoblastic lesion formation *in vivo*

**Figure 5:**
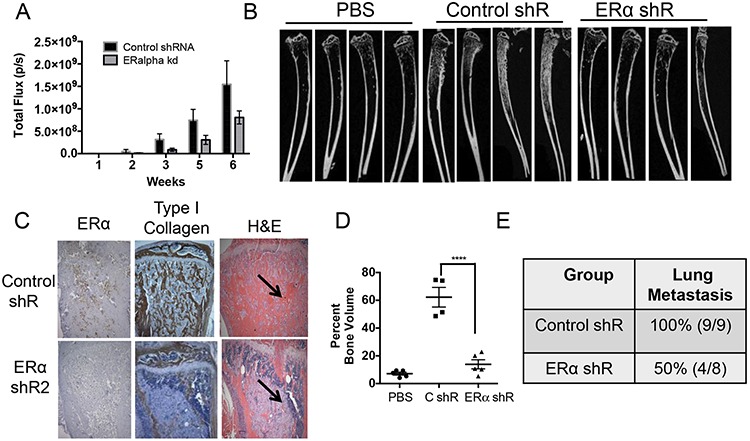
ERα knockdown in PacMetUT1 cells reduces osteoblastic lesion formation and lung metastasis **A.** Total photon flux of intratibia tumors was obtained through whole body bioluminescence imaging of mice inoculated with PacMetUT1 control and ERα knockdown cells. Data are presented as mean ± sem from nine control and eight ERα knockdown mice containing tumors. **B.** Representative micro-CT images are presented for tibiae injected with PBS or PacMet/Luc-GFP cells transfected control or ERα shRNA. **C.** Representative images of immunohistochemical staining of tibia sections for ERα and Type I collagen are presented. Corresponding H&E stained images are also shown. Arrow indicates PacMetUT1 tumor cells that are less stained than the bone tissue. **D.** The total bone volume expressed as percentage of a defined section of tibia volume was plotted from micro-CT imaging data. Data are presented as mean ± sem from 4–5 tibiae in each group. ****P < 0.0001 with one-way ANOVA. **E.** Lung metastasis incidence in the control and ERα knockdown group was confirmed in the excised whole lungs with green fluorescence imaging for GFP positive tumor colonies after the termination of experiment at 7 weeks.

Since estrogen signaling through ERα enhanced the mesenchymal and osteoblast-like features of PacMetUT1 cells, we next examined the role of ERα in osteoblastic tumorigenesis *in vivo*. The control and ERα knockdown PacMetUT1/Luc-GFP cells were injected into the right tibia of 5-week-old male nude mice at 1 × 10^5^ cells/mouse. PBS was injected into the contralateral left tibia as control. Bioluminescence imaging revealed a slower growth rate of intratibia tumors formed by ERα knockdown cells than the control cells although the differences were not significant at all time points (Figure 5A). At the end of 7 weeks after tumor cell inoculation, we harvested the tibiae and lungs to check for bone remodeling and lung metastasis. Micro-CT analysis showed a high intensity of trabecular bone in tibiae injected with cancer cells (Figure 5B) demonstrating the robust osteogenic activity of PacMetUT1 cells as previously published [31]. Furthermore, ERα knockdown in PacMetUT1 led to a remarkable reduction in trabecular bone formation (Figure 5B). We detected a reduced immunostaining of mouse specific type I collagen in the bone matrix (Figure 5C) and a significant reduction in bone volume (Figure 5D) in ERα knockdown tumor cells. The ERα knockdown in PacMetUT1 did not result in changes in AR or ERβ expression in the mouse tibia (Supplementary Figure 2A–2B). These results suggest that ERα expression in the PacMetUT1 cell is essential for its osteoblastic tumorigenic property. Consistent with the notion that EMT promotes metastasis, we detected metastasis to lungs by examining GFP-positive metastatic nodules under an epi-fluorescence microscope as done previously [31]. We observed a significant reduction of 50% (*P* < 0.05, Fisher's Exact Test) in lung metastasis incidence rate in ERα knockdown group in comparison with the control group (Figure 5E).

### Treatment with ERα inhibitor ICI 182,780 inhibits osteoblastic bone formation

**Figure 6:**
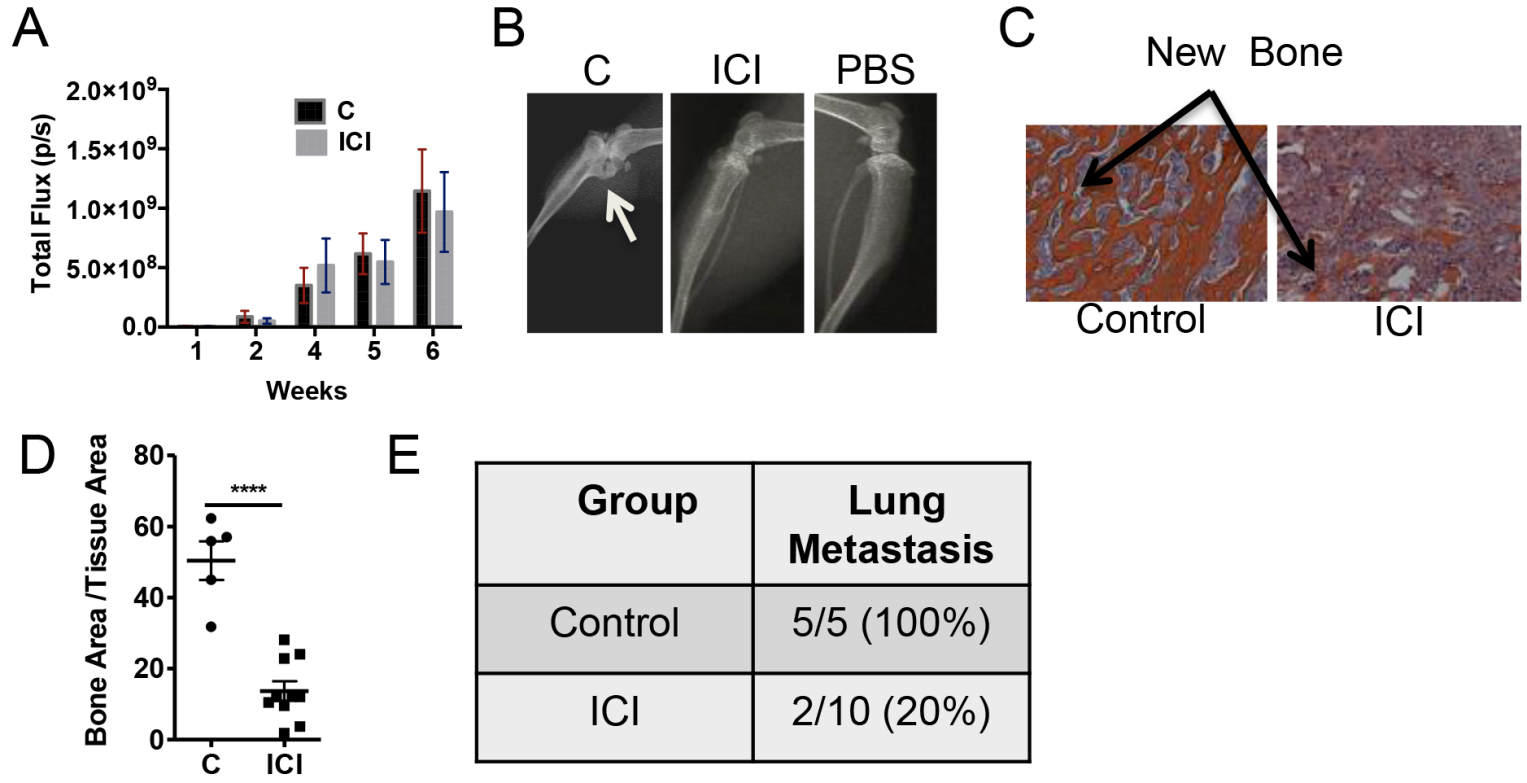
Treatment with pharmacological inhibitor ICI reduces osteoblastic lesion development and lung metastasis **A.** Whole body bioluminescence imaging was performed to measure tumor growth over time in the tibiae. **B.** and **C.** Representative images from X-ray radiography and H&E staining are shown. Arrow shows new bone formation inside the tibia. **D.** Bone area to total tissue area was quantified with BIOQUANT analysis software. Data are presented as mean ± sem with each dot representing one tibia. **E.** Lung metastasis incidence was confirmed in the excised whole lungs after the termination of experiment at 7 weeks. ****P < 0.0001.

As ERα in PacMetUT1 cells contributes to osteoblastic tumor formation, we next investigated the effect of pharmacological inhibition of ERα with ICI 182,780 on osteoblastic lesion formation. The male nude mice were injected with PacMetUT1/Luc-GFP cells in the right tibiae and PBS in the left tibiae, and treated with ICI at 5mg/kg once a week for 7 weeks. There was no change in tumor growth rate in bone with ICI treatment (Figure 6A). However, there was a significant reduction in bone formation in the ICI treatment group with both radiographic imaging (Figure 6B) and histologic staining in comparison with the vehicle treated group (Figure 6C). There was a significant reduction in bone formation in ICI treated group with histomorphometry analysis (Figure 6D). Similar to the ERα knockdown, ICI treatment also significantly (*P* < 0.01, Fisher's Exact Test) reduced lung metastasis incidence to 20% from 100% in the vehicle treated group (Figure 6E). All these results indicate a positive role of ERα in promoting bone formation and lung metastasis in PacMetUT1 cell model *in vivo* and the potential utility of ERα inhibitors for the intervention of osteoblastic lesion formation.

### ERα mediates cross-talk between prostate cancer cells and pre-osteoblasts

**Figure 7:**
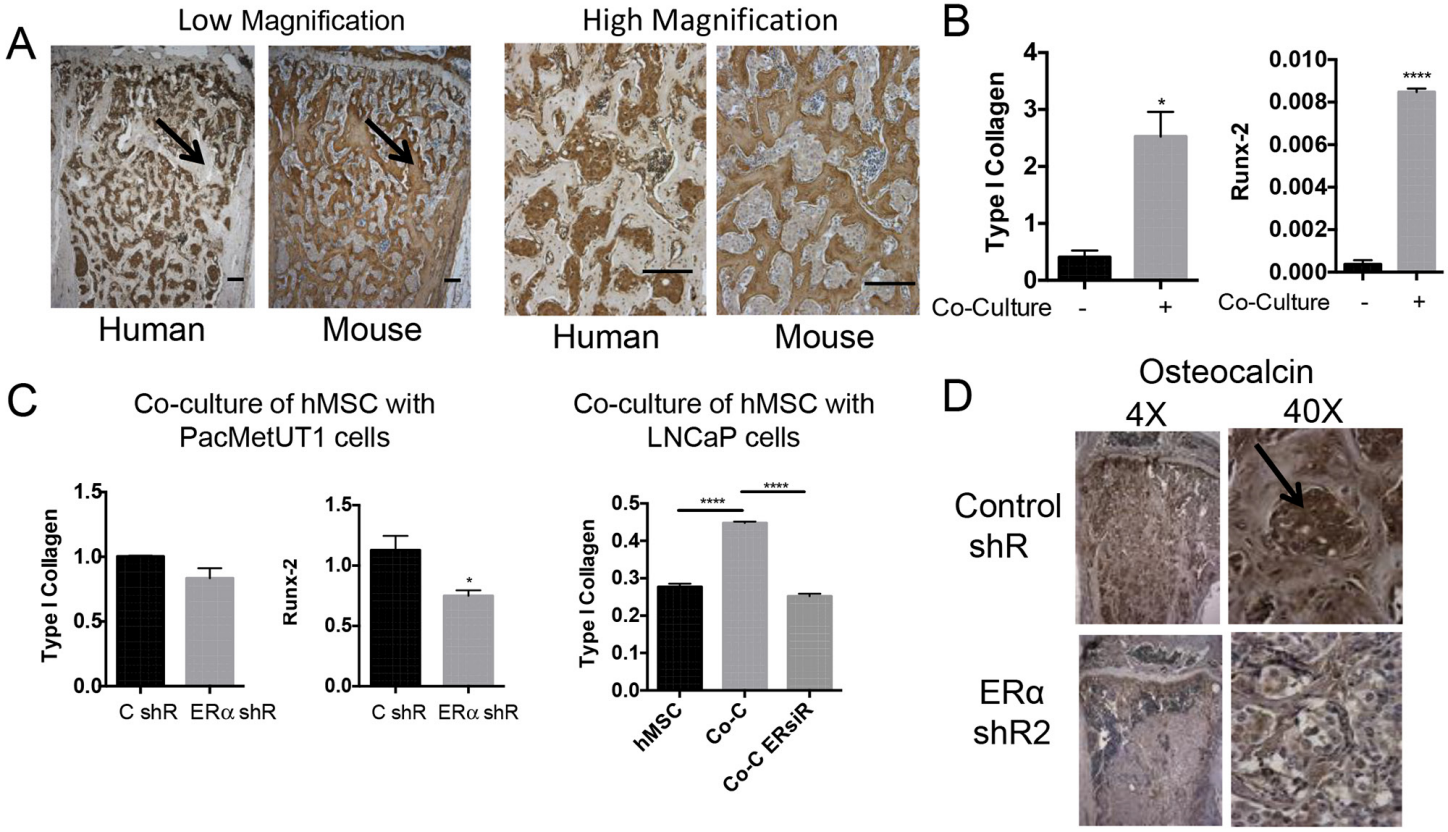
ERα knockdown in prostate cancer cells attenuates their ability to induce osteoblastic marker expression in pre-osteoblasts **A.** Immunohistochemistry with human and mouse Type I Collagen antibody in two consecutive tibia section with PacMetUT1-formed tumor indicates new bone formed is stained only with mouse collagen, while tumor cells are stained with human collagen with little overlapping staining. Scale bar = 200 μm. **B.** Osteogenic markers, Type I collagen and Runx-2, in hMSC cells were measured with real-time RT-PCR after being co-cultured with PacMetUT1 cells for 4 days. **C.** Cells with or without ERα knockdown were co-cultured with hMSC for 4 days. Osteogenic markers were then measured in hMSC with real-time RT-PCR. **D.** Immunohistochemistry for osteoblast marker (osteocalcin) was performed in tibiae injected with control and ERα knockdown PacMetUT1/Luc-GFP cells. Arrow indicates a positive staining. Data in the panel B and C are presented as mean ± sem from triplicate measurements. *P < 0.05, **P < 0.01 and ****P < 0.0001.

The osteomimicry properties of prostate cancer cells led us to examine whether cancer cells themselves could form calcium nodules *in vitro* and trabecular bone *in vivo*. However, we did not observe a positive alizarin red staining for mineralization when PacMetUT1 cells were cultured in mineralization-inducing medium for 3 weeks. This is in contrast to some of the published reports observing a positive staining with other prostate cancer cells [38, 39]. Consistent with our *in vitro* findings, we did not detect a positive staining of human type I collagen in tibiae sections with new trabecular bones after PacMetUT1 injection (Figure 7A). Although PacMetUT1 cells were stained positive with anti-human type I collagen antibody (Figure 7A) suggesting that tumor cells themselves do not form bones. Hence, we tested the possibility that tumor cells could induce pre-osteoblasts to form excessive amount of osseous tissues. Indeed, co-culture of hMSCs with prostate cancer cells upregulated osteogenic markers in hMSCs (Figure 7B). The knockdown of ERα in cancer cells decreased their potency in stimulating osteogenic marker expression in hMSCs under co-culture condition (Figure 7C). Interestingly, there was a reduced expression of osteocalcin in the tibiae injected with ERα knock down PacMetUT1 tumors (Figure 7D). These results suggest that the tumor cells can induce osteoblast differentiation and bone formation by pre-osteoblasts by secreting factors that are regulated by estrogen/ERα.

## DISCUSSION

Metastasis is the primary reason of mortality in cancer. Prostate cancer patients who are in the advanced stage of the disease often develop bone metastasis that is predominantly osteoblastic. The mechanisms of osteoblastic lesion development are not well defined. A variety of factors have been implicated as potential mediators such as endothelin-1 [40], fibroblast growth factors (FGFs), bone morphogenetic proteins (BMPs) [41], and Wnts [42]. Other factors, such as parathyroid hormone-related protein (PTHrP) and RANKL have been reported to mediate osteolytic metastasis of prostate cancer [43]. One major challenge in the field is the lack of model systems that mimics osteoblastic lesions [44, 45]. We have established a novel osteoblastic model *in vivo* using a human prostate cancer derived cell line, PacMetUT1 [31]. In the present study, we took advantage of this system to discover a new role of estrogen and ERα in osteoblastic tumorigenesis.

Estrogens have been considered to be significant risk factors in the development of benign prostatic hyperplasia and prostate cancer. The presence of estrogen receptors in the prostate tissue suggests that estrogens can have functional roles in the prostate. It has been widely reported that ERβ is the predominant subtype, expressed in the majority of prostate epithelial cells, whereas ERα is expressed typically in the prostate stromal cells. ERα-positive cells have also been found in the hyperplastic epithelium of the prostatic ducts, but its functional role remains undefined [46]. Studies by Bankoff et al. showed ERα expression in prostate cancer and in premalignant prostatic lesions [27]. Due to our limited understanding of ERα contribution in prostate cancer, we investigated the role of estrogen and ERα in prostate cancer malignancy. ERα is expressed in prostate cancer cells at varying levels with PacMetUT1, C4–2, 22Rv1 and LNCaP cells expressing higher level of ERα compared to BPH-1. We further found that estrogen had an oncogenic effect on prostate cancer cells by increasing cell migration and anchorage-independent growth. It was shown previously that stimuli such as TGFβ and hypoxia could inhibit ERβ expression in prostate cancer cells, and the loss of ERβ promoted EMT [33]. We observed a down-regulation in E-cadherin and an increased Snail and Vimentin expression in prostate cancer cells with estrogen treatment. The knockdown of ERα in PacMetUT1 abrogated these effects of estrogen suggesting that the estrogen induced EMT is mediated by ERα. To further check the role of ERα in osteoblastic lesion formation, we injected the control and ERα knockdown PacMetUT1 cells directly in the tibia of male nude mice. The micro-CT analysis of tibiae sections showed significant reduction in bone formation by the PacMetUT1 cells when ERα was knocked down suggesting that ERα expression in PacMetUT1 is essential for osteoblastic tumor formation *in vivo*. Systemic treatment of mice with an ERα inhibitor, ICI 182, 780, also significantly reduced osteoblastic tumor formation. These results suggest that the blockade of ERα signaling may prevent prostate cancer-induced bone impairment and maintain bone health. This notion is consistent with a recent phase III clinical trial using a selective estrogen receptor modulator Toremifene in prostate cancer demonstrating a significant decrease in the incidence of new vertebral fractures in men receiving androgen deprivation therapy for prostate cancer [11].

Some studies suggest that tumor cells can survive and proliferate in bone tissues by acquiring a bone-cell phenotype and osteoblastic features termed osteomimicry [36, 47]. We also observed expression of osteogenic markers and alkaline phosphatase activity when prostate cancer cells were cultured in osteogenic differentiation medium. Estrogen enhanced the increase in osteogenic markers whereas ERα knockdown attenuated the osteoblast-like features in prostate tumor cells. However, analysis of tibiae sections in PacMetUT1 osteoblastic *in vivo* model revealed staining of newly formed bone tissues with mouse collagen antibody. This suggests that the human prostate cancer cells themselves did not form bones, but likely induced the differentiation of mouse pre-osteoblasts. Indeed, co-culture of human osteoblast precursor cells (hMSC) with prostate cancer cells induced osteoblastic marker expression in hMSCs. Inhibition of ERα in prostate cancer cells further abrogated this effect. Further studies are required to delineate the key factors involved in the tumor-bone stroma crosstalk that is affected by estrogen/ERα signaling. Since PacMetUT1 has unique features in expressing low AR and high levels of ERα, we queried the cancer genome atlas database (TCGA) to check how many tumor samples have similar expression patterns. Out of 419 tumor samples, we found that 43 samples have high levels of ERα compared to AR, which represents about 10.26% of the total tumor population examined (supplementary Figure 3A–3G).

Pulmonary metastasis commonly develops in prostate cancer patients who have bone metastasis [48]. We found a significant reduction in lung metastasis *in vivo* with both genetic and pharmacological inhibition of ERα in tumor cells. This could be due to the reduced ability of ERα knockdown PacMetUT1 to invade and migrate into the bone marrow as our *in vitro* studies suggested that the blockade of ERα signaling inhibited EMT, which is known to promote tumor cell migration and invasion.

In summary, our study revealed a novel role of estrogen signaling in promoting osteoblastic tumor formation in human prostate cancer cell lines. This oncogenic role of estrogen is mediated by ERα. Thus, inhibition of ERα signaling in prostate cancer cells may be a novel therapeutic strategy to inhibit the osteoblastic lesion development in patients with advanced stage prostate cancer.

## MATERIALS AND METHODS

### Cell cultures

LNCaP, 22Rv1, MDA-MB-231, MCF-7 and MDA-PCa-2b cell lines were purchased from American Type Culture Collection (ATCC, Manassas, VA, USA). These cells were cultured in medium recommended by ATCC. BPH-1 cell line was obtained from Dr. Scott Lucia's laboratory and cultured in a RPMI1640 medium containing 10% fetal bovine serum. PacMetUT1 was isolated from the lymph node metastasis of a 57-year old prostate cancer patient at our university [49]. Freshly isolated human bone marrow mononuclear cells, containing BM-MSCs (primary cells) from 20–25 year old donors, were purchased from Lonza (Walkersville, MD) and cultured in alpha-minimum essential medium (MEM; Gibco BRL, Life Technologies, NY, USA) supplemented with 15% fetal bovine serum (FBS, Gibco). All the cells were maintained at 37°C in a 5% CO_2_ humidified incubator.

### Chemicals

Estrogen (17β-estradiol) and tamoxifen were purchased from Sigma (St. Louis, MO, US), ICI 182,780, 1,3-Bis(4-hydroxyphenyl)-4-methyl-5-[4-(2-piperidinylethoxy)phenol]-1H-pyrazole dihydrochloride (MPP dihydrochoride), and 4,4′,4′'-(4-Propyl-[1H]-pyrazole-1,3,5-triyl)trisphenol (PPT) were purchased from Tocris Bioscience (Bristol, United Kingdom).

### *In Vitro* luciferase assay

For transient transfection, cells were seeded in triplicates in a 12-well plate at a density of 1.5–2.0 × 10^5^ cells/well in the culture medium containg 10% charcoal-stripped fetal bovine serum. When the cultures were 80% confluent, they were co-transfected with 500 ng of an estrogen responsive promoter-luciferase construct (ERE-Luc) and 100 ng of a β-galactosidase expression plasmid using 1.8 μL of Lipofectamine 2000 (Invitrogen, Carlsbad, CA, USA). After 5 hours, the medium was replenished containing estrogen (10 nM) or vehicle. Luciferase assay was performed as described previously [31, 50] after 24 hour of incubation.

### Cell proliferation assay

Cells were plated in 96-well plate at 2,000 cells per well in five-well replicate. After 4 hours, cells were treated with different chemicals for 5 days. MTT assays were used for quantifying numbers of viable cells with a Biotek plate reader (Biotek Instruments, Winooski, VT, US) as described previously [31].

### Western blot analysis

Cells after harvesting were lysed in Laemmli buffer containing protease inhibitors and processed as described previously [31]. The antibodies to ERα and ERβ were purchased from Millipore, to GAPDH from Calbiochem (Billerica, MA, US), to actin from Sigma (St. Louis, MO, US), to E-cadherin from BD Biosciences (San Jose, CA, US), and to Snail from Abcam (Cambridge, MA, US).

### Alkaline phosphatase activity assay

Cells were seeded in 12-well plates, proliferation and osteogenesis induction medium (Chemicon, EMD Millipore, Billerica, MA, USA), which consisted of 0.1 μM dexamethasone, 0.2 mM ascorbic acid-2-phosphate and 10 mM Glycerol-2-phosphate. Medium was replenished every 2 days. ALP activity was measured after 5 days using Quantichrom alkaline phosphatase assay kit (DALP - 250, BioAssay Systems, Hayward, CA, USA).

### Animal experiments

Four- to five-week old male athymic nude mice were purchased from Harlan Sprague-Dawley, Inc. (Indianapolis, IN, US). The animal protocol was approved and monitored by the Institutional Animal Care and Use Committee. Animals were housed under the care and supervision of Laboratory Animal Research Facility at the University of Texas Health Science Center, San Antonio, Texas, US.

### Intratibia injections

Animal surgery was performed under constant anesthesia with isofluorane inhalation. PacMetUT1 cells (1 × 10^5^ in 10 μL PBS) were inoculated into the bone marrow area of right tibiae through a pre-made hole using a Hamilton syringe fitted with 27-gauge needle as described previously [31]. The left tibiae were injected with PBS as controls for the surgery.

### Bioluminescence imaging analysis

After mice were anesthetized with isofluorane inhalation, D-luciferin (Xenogen) was injected i.p. at 75mg/Kg body weight in PBS with a 1 min interval for each mouse. Ten minutes later, bioluminescence images were acquired with IVIS spectrum Imaging system (Xenogen) at 1 min interval for each mouse as described previously [31].

### Radiographic analysis

Mice were exposed with an X-ray at 35 KVP for 5 sec by using a Faxitron Digital Radiographic Inspection unit against the detector as described [51].

### Bone histomorphometry analysis

Bone tissues were fixed in 70% ethanol for 24–48 h at room temperature, decalcified in 10% EDTA, and embedded in paraffin. Sections were stained with hematoxyoin and esosin, orange G and phloxine. The trabecular bone and tumor areas in a tibia section were examined under a Nikon Eclipse E800 microscope equipped with a QImaging QICAM-F fast color digital camera and quantified using Bioquant Osteo System (Bioquant Image Analysis Corporation, Nashville, TN, US) as was described previously [31].

### MicroCT analysis

Micro-Computed tomography (μCT) was performed on the tibia using a desktop SkyScan 1172 (Bruker, Aartselaar, Belgium) system. Each specimen was submerged in 70% ethanol, positioned with the proximal tibia pointing upward and the tube was sealed with parafilm. Samples were scanned at 60 kV, 167 μA beam intensity, 10 μm image pixel size, 0.7° rotation step, 4 frames averaging, and a 700-millisecond exposure time at each step. The structural properties of the trabecular bone were evaluated in the proximal metaphysis. The volume of interest (VOI) started 0.5 mm distally from the proximal growth plate and continued distally for 1.5 mm. The trabecular boundary was outlined manually. 3D morphometric analyses were performed on the trabecular VOIs.

### RNA isolation and real time PCR analysis

Cells were lysed in TRIzol reagent (Sigma) for dissociation of any RNA-protein complexes and RNA was isolated as previously described [52]. Primer sequences are available upon request.

### Co-culture assay

Human mesenchymal stem cells were seeded in 24-well plates, grown for 24 h, and then co-cultured with or without control and ERα knockdown PacMetUT1/Luc-GFP, 22Rv1 or LNCaP cells in cell culture inserts (1.0-μm pore size, Becton Dickinson, Durham, NC, USA). After 4 days of co-culture, inserts containing prostate cancer cells were removed and total RNA was isolated from hMSC cells for quantitative real-time PCR analysis.

### Migration assay

Cells were seeded in the upper 24-well Boyden chambers with 8 micron-size inserts (BD Biosciences, Durham, NC, USA) with or without treatments in serum-free containing medium. Serum-containing medium was added to the lower chamber. Cell migration was counted after 18 hours by fixing and staining the cells with Hema 3 Stain 18 kit (Fischer Scientific, Waltham, MA, USA) according to the manufacturer's protocol.

### Immunohistochemistry

Bone tissue sections were rehydrated through xylene and graded ethanol, incubated in the proteinase K (20 μg/ml) for 10 min and then sections were blocked for endogenous peroxidase with 3% hydrogen peroxide (Thermo Fisher Scientific, Waltham, MA, USA) for 30 min in room temperature. Sections were permeabilized and blocked in 10% goat serum for 1 hr. The primary antibodies were anti-collagen I (1:600, ab138492 and ab21286, Abcam, Cambridge, MA, USA) diluted in the 5% goat serum and incubated at 4°C overnight. Sections were then incubated with a biotinylated goat anti-rabbit antibody (BD Pharmingen, San Diego, CA, USA). For detection, Streptavidin-Horseradish Peroxidase and DAB Substrate Kit (BD Pharmingen, San Diego, CA, USA) were used and the counterstain was done with hematoxylin. For osteocalcin (AbD Serotec 7060–1815, 1:1200 dilution), androgen receptor (Santa Cruz, sc-816, 1: 200) and ERβ (Santa Cruz, sc-8974) antigen retrieval was done with 0.5% trypsin at 37°C for 30 min and for estrogen receptor alpha (Santa Cruz 8002, 1:150), antigen retrieval was done in 10 mM sodium citrate, pH 6, at 95°C for 15 min.

### The cancer genome atlas database (TCGA) data analysis

Level3 data of gene expression reads count estimated by RSEM [53] of 419 tumor and 52 normal samples of prostate adenocarcinoma (PRAD) in TCGA were downloaded. Reads Per Kilobase of transcript per Million mapped reads (RPKM) values of gene *g* in sample *s* were calculated from estimated reads counts by the modified formula below [54]:

$${\text{RPKM}}_{g,s}=\frac{{\text{ReadCount}}_{g,s}\times 10^{9}}{\left(\Sigma_{g\;\mathrm{in}\;s}{\text{ReadCount}}_{g,s}\right)\times {\text{GeneLength}}_{g}}$$

Gene lengths are the summed lengths of reduced exons from annotation package Homo.sapiens in Bioconductor/R. Log2 (RPKM + 1) values were used as Log2 Expression values to compare gene expression across samples. Gene expression values of ESR1, ESR2 and AR were used for generating Heatmaps using heatmap.2 from package gplots/R. Sample distance were calculated in Pearson Correlation and hierarchical clustering were performed in complete linkage method. Significance of difference between gene expression levels of ESR1 and AR were called based on cutoff at one-tail *p* value 0.1 on the transformed z scores.

### Statistical analysis

Two-tailed Student's *t*-test was used to compare two groups. One-way analysis of variance was used for analyzing data when more than two groups were used with Tukey-Kramer post hoc test. Results are expressed as mean ± sem. *P* < 0.05 was considered to be statistically significant.

## SUPPLEMENTARY FIGURES


