# Supplementary material for: Estrogen and estrogen receptor alpha promotes malignancy and osteoblastic tumorigenesis in prostate cancer
Source: Oncotarget. 2015 Oct 31;6(42):44388–402. doi: 10.18632/oncotarget.6317 (PMC4792564; doi:10.18632/oncotarget.6317)
Supplement: Supplementary file 1 [file oncotarget-06-44388-s001.pdf]

SUPPLEMENTARY FIGURES

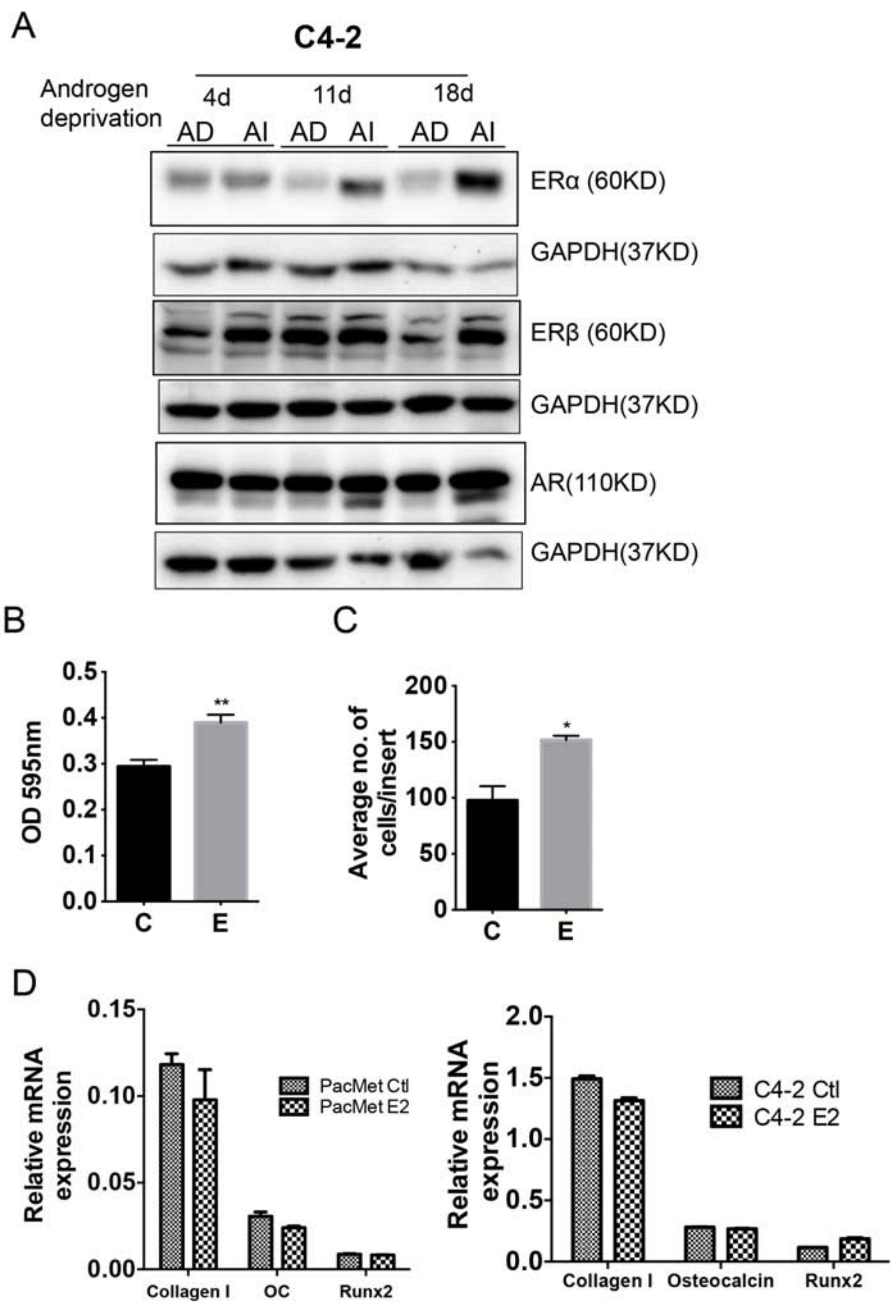

Supplementary Figure S1: Estrogen increases anchorage-dependent cell growth and migration in C4–2 cells. A. Cell lysates from C4–2 cells adapted in androgen-deprived medium (AI) for 4, 11 and 18 days successively were used for ERα, ERß and AR expression analysis by Western blot. GAPDH was used as an equal loading control. AD stands for growth in normal serum medium, which was not deprived of androgen. **B.** C4–2 cells were plated (2,000 cells/well) and treated with estrogen (10 nM) for 5 days. Cell growth was measured by MTT assay. Data presented are mean ± sem from six measurements. $^{**}P < 0.01$ using two-tailed student's *t*-test. **C.** Migration of C4–2 cells (40,00 cells/well) after treatment with estrogen (10 nM) was counted in whole inserts after 9 h. Data presented are mean ± sem from triplicate wells. * $P < 0.05$ using two-tailed student's *t*-test. **D.** Osteogenic markers (Type I Collagen, Osteocalcin and Runx-2) were measured by real-time RT-PCR in PacMetUT1 and C4–2 cells after treatment with estrogen (10nM) in the proliferation medium for 5 days. Data presented are mean ± sem from triplicate measurements.

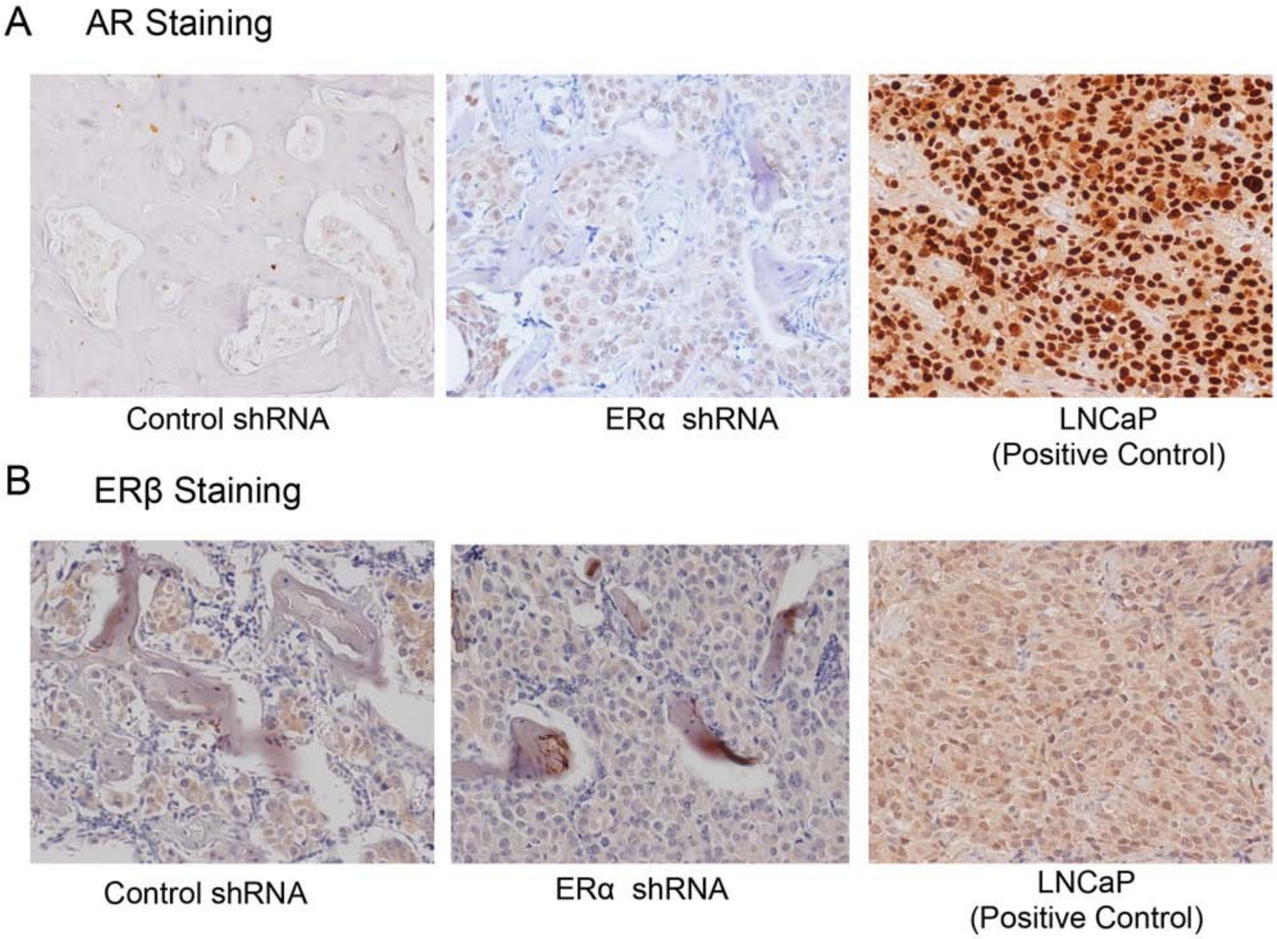

Supplementary Figure S2: AR and ERβ staining in bone samples after ERα blockade. A and **B.** Immunohistochemistry with human AR and ERβ antibodies was performed in tibia sections with tumors formed by control or ERα knockdown PacMetUT1 cells. Tumor sections from LNCaP cell-formed subcutaneous tumors were used as positive controls for both antibody staining.

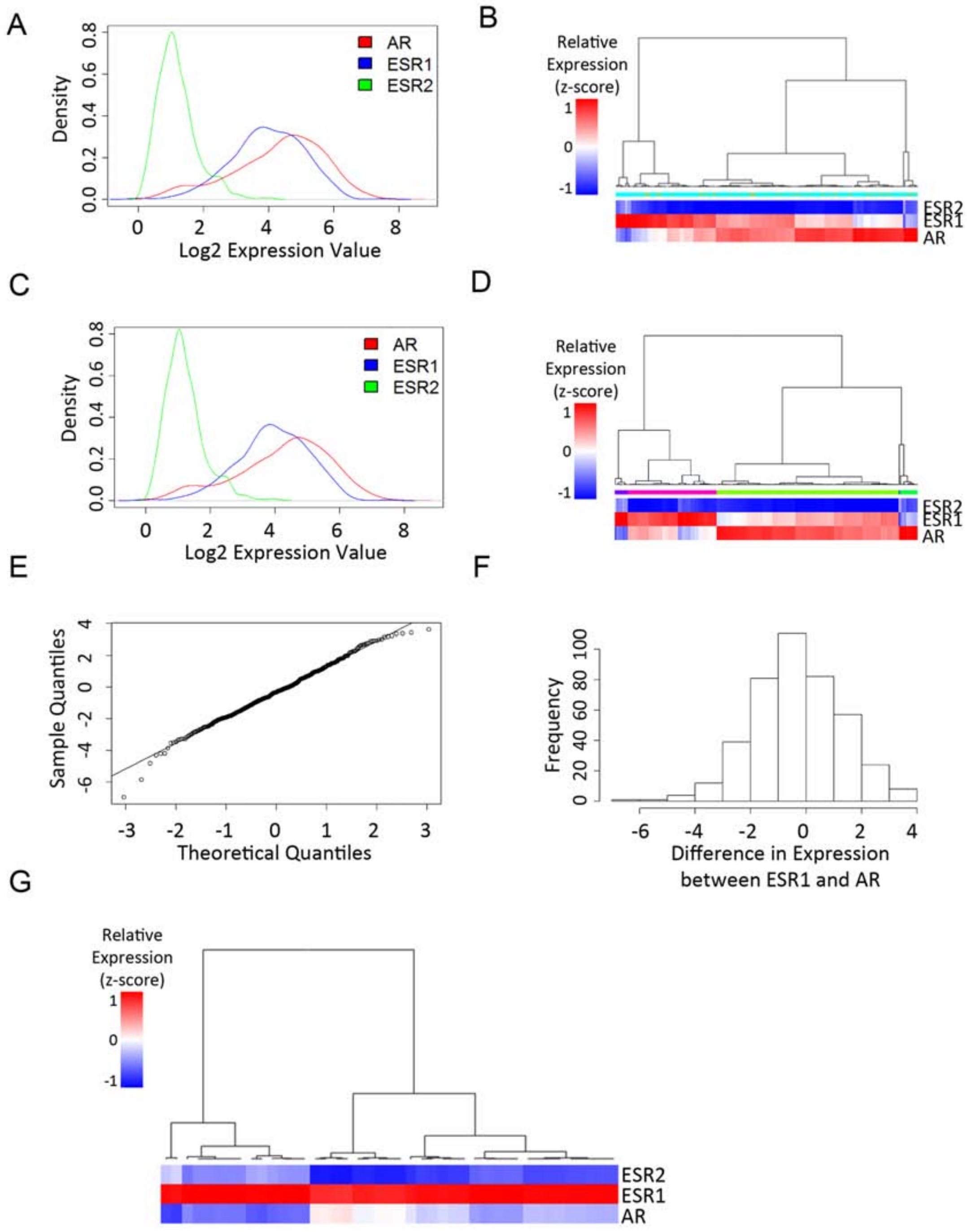

Supplementary Figure S3: TCGA data analysis of AR, ERα and ERβ analysis in normal and prostate cancer samples. A. Frequency distribution of expression levels of genes ESR1, ESR2 and AR in 419 tumor and 52 normal samples. **B.** Heatmap showing the relative expression levels of genes ESR1, ESR2 and AR in 419 tumor and 52 normal samples. Column side color bar indicates sample types are tumor (blue) or normal (orange). **C.** Frequency distribution of expression levels of genes ESR1, ESR2 and AR in 419 tumor samples. **D.** Heatmap showing the relative expression levels of genes ESR1, ESR2 and AR in 419 tumor samples. Column side color bar indicates hierarchical clustering of tumor samples based on expression levels of three genes. Cluster in pink indicates samples where ESR1 is higher expressed than AR. **E.** Quantile plot shows the differences between expression levels of ESR1 and AR follow normal distribution. **F.** Histogram of the differences between expression levels of ESR1 and AR. Positive values indicate that ESR1 expression is higher than AR expression in those samples. **G.** Heatmap showing the relative expression levels of genes ESR1, ESR2 and AR in 43 tumor samples where expression level of ESR1 is significantly higher than AR.
